# Unaddressed Challenges in the Treatment of Cutaneous Melanoma?

**DOI:** 10.3390/medicina60060884

**Published:** 2024-05-28

**Authors:** Alessia Villani, Luca Potestio, Aimilios Lallas, Zoe Apalla, Massimiliano Scalvenzi, Fabrizio Martora

**Affiliations:** 1 Section of Dermatology, Department of Clinical Medicine and Surgery, University of Naples Federico II, 80131 Naples, Italyfabriziomartora92@libero.it (F.M.); 2 First Department of Dermatology, School of Medicine, Faculty of Health Sciences, Aristotle University, 54124 Thessaloniki, Greece; alallas@auth.gr; 3 Second Department of Dermatology, School of Medicine, Faculty of Health Sciences, Aristotle University, 54124 Thessaloniki, Greece

**Keywords:** melanoma, management, treatment, unaddressed challenges, personalized medicine

## Abstract

*Background and Objectives*: While the management of noninvasive cutaneous melanoma (CM) is typically limited to a secondary excision to reduce recurrence risk and periodic follow-up, treating patients with advanced melanoma presents ongoing challenges. *Materials and Methods*: This review provides a comprehensive examination of both established and emerging pharmacologic strategies for advanced CM management, offering an up-to-date insight into the current therapeutic milieu. The dynamic landscape of advanced CM treatment is explored, highlighting the efficacy of immune checkpoint inhibitors and targeted therapies, either in monotherapy or combination regimens. Additionally, ongoing investigations into novel treatment modalities are thoroughly discussed, reflecting the evolving nature of melanoma management. *Results*: The therapeutic landscape for melanoma management is undergoing significant transformation. Although various treatment modalities exist, there remains a critical need for novel therapies, particularly for certain stages of melanoma or cases resistant to current options. *Conclusions*: Consequently, further studies are warranted to identify new treatment avenues and optimize the utilization of existing drugs.

## 1. Introduction

Cutaneous melanoma (CM) is the most common form of melanoma, deriving from pigment-producing cells called melanocytes, normally found in skin, leptomeninges, inner ear and eye [1,2,3]. Its incidence is increasing, with more than 200,000 new diagnoses per year [1,2]. Moreover, with an overall mortality of about 20%, CM is responsible for more than 80% of skin cancer-related deaths [4,5]. As regards CM pathogenesis, it is multifactorial and not fully understood. However, several risk factors have been identified, such as sunburn, ultraviolet radiation, personal or familiar history of CM, and phenotypic features [6,7,8].

Clinically, four CM subtypes are most common: superficial spreading melanoma (41%), nodular melanoma (16%), lentigo maligna melanoma (2.7–14%) and acral lentiginous melanoma (1–5%) [9,10]. However, other uncommon subtypes can be identified, such as spitzoid melanoma, amelanotic melanoma, desmoplastic melanoma, etc. [5,6,11].

Tumor staging at the diagnosis is the main predictor factor for survival rate, ranging from 98.3% to 16% at 5 years for localized and metastatic melanoma, respectively [12,13].

Four stages can be distinguished: localized disease (stage I and II), locally advanced disease with lymph nodes involved (stage III), and metastatic disease (stage IV) [12,13]. The staging of the primary lesion is based on vertical tumor thickness (Breslow’s depth), presence of ulceration, and mitotic rate [10,11]. Tumor staging is based on vertical tumor thickness (Breslow’s depth), presence of ulceration, and mitotic rate [12,13,14,15].

Fortunately, about 90% of melanoma are diagnosed as primary cancers in early stage, without metastases detected. 

As regards the management, current guidelines suggest surgical excision with safety margins, based on Breslow’s thickness, as the mainstay of treatment, reserving lymph node biopsy if tumor thickness is >0.8 mm [12,16]. In this context, despite the management of noninvasive CM melanoma being limited to a secondary excision to reduce the risk of recurrence and periodic follow-up, the treatment of patients with advanced melanoma is often challenging, due to the ability of cancer cells to escape from the immune system [12,16]. Historically, the use of chemotherapies has been the mainstay for these forms of cancer, with poor outcomes, mainly due to melanoma resistance to apoptosis [12,16]. Recently, new knowledge on melanoma pathogenesis has led to the development of effective treatments, and several drugs are currently under investigation [12,16]. Currently the main options for advanced/metastatic disease are immunotherapy and targeted therapies.

However, several limitations may make these treatments ineffective or inadequate for cancer resistance, patient comorbidities or toxicities [17,18]. In this scenario, we performed a review of the literature with the aim of providing a complete overview on currently available and emerging treatments for melanoma, in order to offer readers a wide perspective on the therapeutic scenario, as well as try to answer the unresolved challenges in melanoma management.

## 2. Materials and Methods

A comprehensive review of the existing literature was conducted by utilizing the primary databases (Embase, PubMed, Cochrane Skin, and clinicaltrials.gov) up to 15 December 2023. The search encompassed key terms such as “cutaneous melanoma”, “invasive melanoma”, “surgical interventions”, “sentinel lymph node biopsy”, “immunotherapy”, “checkpoint inhibitors”, “targeted therapies”, including specific agents like “ipilimumab”, “pembrolizumab”, “nivolumab”, “BRAF inhibitors”, “vemurafenib”, “dabrafenib”, “MEK inhibitors”, “binimetinib”, and “trametinib”, among others. Additionally, terms related to treatment modalities such as “sequential therapy”, “vaccines”, “anti-vascular endothelial growth factor”, “cytokines”, “inhibitory molecules”, and “T-cell agonists” were also included.

Reviews, meta-analyses, clinical trials, real-life studies, case series, and case reports, were analyzed. Articles focusing on treatments for non-advanced CM were excluded.

It is important to note that only articles in English were considered, with manuscripts in other languages being excluded. Furthermore, this article is a synthesis of previously conducted studies and does not involve any original research with human participants or animals conducted by any of the authors.

## 3. Results

The results collected in this review included data for every treatment available for melanoma, from surgery to sentinel lymph node, from approved treatments to those that are still being studied.

### 3.1. Surgical Treatment

Surgery is the gold standard treatment of resectable CM. Current guidelines recommend peripheral surgical margins according to tumor thickness, as follows: 0.5 mm for melanoma in situ, 1 cm for CM with a Breslow thickness < 1 m; 1–2 cm margins for CM with a Breslow thickness < 2 mm, >2 cm margins in case of Breslow thickness > 2 mm [19]. Notably, Mohs micrographic surgery may be used in some cases, such as those of lentigo maligna or cancers affecting sensitive areas, in order to ensure complete resection with reduced margins [18,19,20,21].

### 3.2. Sentinel Lymph Node Biopsy

The current guidelines advocate for sentinel lymph node biopsy (SLNB) in patients with CM that is thicker than 1 mm. Additionally, SLNB is recommended for lesions with a thickness of 0.8 mm if histological examination has identified additional risk factors such as ulceration, presence of ≥1 mitosis/mm^2^, and/or lymphovascular invasion. This recommendation holds particular importance in cases involving younger patients [17]. If SLNB is negative, no further actions are required while adjuvant treatment replaces the complete lymph node dissection in case of lymph nodal involvement [17].

### 3.3. Systemic Treatments

Neoadjuvant therapy is the first treatment used to reduce the tumor before surgery [17,22]. In particular, stage III of melanoma can be considered in regard to neoadjuvant therapy [17,22]. Despite the numerous clinical trials on the use of neoadjuvant therapy in CM treatment that are ongoing, data are limited. However, preliminary results show that immunotherapy seems to be superior to targeted therapy [17,22]. In this context, a phase II trial involving 23 patients with high-risk resectable melanoma has demonstrated that the combination therapy of ipilimumab (anti-cytotoxic T lymphocyte-associated antigen-4, CTLA-4) along with nivolumab (anti-programmed cell death protein-1, PD-1) yields superior outcomes when compared with the use of a single-agent anti-PD-1 treatment [22].

Adjuvant therapy is used in combination with surgery to improve survival outcomes [23]. In the past, interferon-alpha was used in patients with a melanoma > 1.5 mm. However, several safety issues were detected [23]. To date, newly approved drugs like immune checkpoint inhibitors (PD-1 and its ligand (PDL-1) and anti-CTLA-4) and targeted therapies (BRAF/MEK inhibitors) that are more effective and safer than interferon-alpha [23,24] have been developed. If BRAFV600 E/K mutation is detected the adjuvant therapy with BRAF/MEK inhibitor can be used in patients with lymph node involvement, while it is important to note that anti-PD-1 can also be used without mutation involved [23].

Finally, as regards metastatic melanoma, three types of metastases deriving from CM can be distinguished, as follows: where lesions have occurred within 2 cm of the primary tumor (satellite metastases), where lesions have occurred more than 2 cm from the primary tumor but are not beyond the regional nodal basin (in-transit metastases), and distant metastases. For metastatic forms of the disease, several systemic therapies are available. Currently approved drugs for advanced melanoma are reported in Table 1.

#### 3.3.1. Immune Checkpoint Inhibitors

Immune checkpoint inhibitors act on small proteins expressed by immune cells and cancer cells called “checkpoints”. These “checkpoints” actively suppress T cells, inducing a dysfunctional state and promoting tumor growth and metastasis [24,25,26,27]. To date, immune checkpoint inhibitors are used for the treatment of several cancers, including melanoma. Blocking the PD-1/PD-L1 and CTLA-4 pathways in melanoma has been shown to be an effective weapon in melanoma management, thanks to the reinvigoration of anti-tumor T cell [24,25,26,27].

##### Nivolumab

Nivolumab is an immune checkpoint inhibitor licensed as monotherapy or in combination with ipilimumab for the treatment of advanced (unresectable or metastatic) melanoma in adults and adolescents 12 years of age and older [28,29]. Regarding adjuvant treatment of melanoma, it can be used as monotherapy for adults and adolescents 12 years of age and older with Stage IIB or IIC melanoma or in patients with involvement of lymph nodes or metastatic disease who have undergone complete resection [28]. The dosage is scheduled as intravenous infusion at a dosage of 240 mg every 2 weeks (Q2W) or 480 mg every 4 weeks (Q4W) as monotherapy or in combination with ipilimumab (nivolumab 1 mg/kg plus ipilimumab 3 mg/kg every 3 weeks (Q3W) for up to four doses, followed by nivolumab as single agent at standard dosage of 240 mg Q2W or 480 mg Q4W) [28].

The CHECKMATE-037 study showed that nivolumab is superior to chemotherapy in patients with BRAF V600 mutation positive metastatic melanoma, previously treated with ipilimumab and BRAF inhibitor [30,31]. These results were also confirmed in previously untreated patients (CHECKMATE-066) [32]. Finally, CHECKMATE-067 showed nivolumab to be superior to ipilimumab in untreated patients with unresectable or metastatic melanoma [32].

##### Pembrolizumab

Pembrolizumab, an anti-PD-1 medication, has received approval for managing metastatic or unresectable melanoma, as well as for adjuvant treatment in melanoma patients. The recommended dosage is either 200 mg every 3 weeks (Q3W) or 400 mg every 6 weeks (Q6W) [33,34].

The KEYNOTE-006 study demonstrated the superiority of pembrolizumab over ipilimumab in previously untreated patients with metastatic or unresectable melanoma. Similarly, findings from the KEYNOTE-002 study revealed that pembrolizumab outperformed chemotherapy in patients with previously treated metastatic or unresectable disease [35,36].

Lastly, results from the KEYNOTE-054 study showcased a significant enhancement in recurrence-free survival (RFS) among patients receiving pembrolizumab as adjuvant therapy compared with those on placebo [37].

##### Ipilimumab

Ipilimumab is a fully human anti-CTLA-4 monoclonal antibody (IgG1κ) [38,39]. Ipilimumab is approved as monotherapy or in combination with nivolumab in the treatment of advanced (unresectable or metastatic) melanoma in adults and adolescents 12 years of age and older [38]. Dosage is 3 mg/kg Q3W for up to four doses [38]. It is also approved for renal cell carcinoma, non-small cell lung cancer (NSCLC), malignant pleural mesothelioma (MPM), mismatch repair deficient (dMMR) or microsatellite instability-high (MSI-H) colorectal cancer (CRC) and oesophageal squamous cell carcinoma (OSCC) [38]. The CA 184-029 study has reported that ipilimumab was significantly superior to a placebo as adjuvant treatment in terms of regression-free survival (RFS) [38]. Combination of nivolumab 1 mg/kg plus ipilimumab 3 mg/kg is approved as a first-line treatment of patients with advanced melanoma [38]. Notably, a recent phase IIIb/IV trial (CHECKMATE 511) enrolled 360 patients with previously untreated, unresectable stage III or IV melanoma and randomized them 1:1 to receive nivolumab 1 mg/kg plus ipilimumab 3 mg/kg or nivolumab 1 mg/kg plus ipilimumab 1 mg/kg. The trial showed a lower incidence of treatment-related grade 3–5 AEs in the second group as compared with the first [40]. Moreover, no differences for any efficacy end point were highlighted, suggesting that a reduced dose of ipilimumab may be more tolerated without losing effectiveness [40]. However, the follow-up of the study was limited to 12 weeks [40].

#### 3.3.2. Target Therapy

Target therapy or molecular target therapies are made up of drugs that affect particular molecular targets present on tumor cells, recognized as fundamental for the growth and uncontrolled proliferation of the tumor cells themselves [41,42]. Activated BRAF-mutated kinase can be inhibited by BRAF inhibitors, and its downstream MEK activation can be inhibited by MEK inhibitors [41,42]. The combined use of BRAF and MEK inhibitors may delay the emergence of drug resistance against BRAF inhibitors [41,42]. Activation of mutations in BRAF (V600E/K) occurs in approximately 50% of cutaneous melanomas, with these tumors vulnerable to BRAF/MEK inhibition [41,42]. In patients with advanced melanomas harboring BRAF V600 mutation, three different combinations of BRAF plus MEK inhibitors have been shown to yield superior clinical outcomes over BRAF inhibitor alone [41,42]. Of note, despite being used in combination, the BRAF and MEK inhibitors are reported as separate drugs.

##### Dabrafenib

Dabrafenib is a kinase inhibitor approved as monotherapy or in combination with trametinib for the treatment of adult patients with unresectable or metastatic melanoma with a BRAF V600 mutation [43]. Regarding adjuvant treatment, this is used in combination with trametinib for adult patients with stage III melanoma with a BRAF V600 mutation, following complete resection. The recommended dose of dabrafenib, either used as monotherapy or in combination with trametinib, is 150 mg (two 75 mg capsules) twice daily (corresponding with a total daily dose of 300 mg) [43]. The recommended dose of trametinib, when used in combination with dabrafenib, is 2 mg once daily [40]. It can also be used in combination with trametinib for the treatment of adult patients with advanced NSCLC with a BRAF V600 mutation [43]. The BREAK-3 study reported that dabrafenib was superior to chemotherapy in melanoma management [44]. The COMBI-AD study showed that dabrafenib plus trametinib is superior to placebo as an adjuvant therapy [45].

##### Trametinib

Trametinib is a kinase inhibitor targeting MEK1 and MEK2 [46]. It is approved in combination with dabrafenib for the treatment of adult patients with unresectable or metastatic melanoma with BRAF V600 mutation [46]. The recommended dose of trametinib is 2 mg once daily [46]. Regarding adjuvant setting, trametinib is indicated in combination with dabrafenib for the adjuvant treatment of adult patients with Stage III melanoma with a BRAF V600 mutation, following complete resection. It is also approved for NSCLC with a BRAF V600 mutation [46]. The recommended dose of dabrafenib, when used in combination with trametinib, is 150 mg twice daily [46]. A METRIC study has reported that trametinib is superior to chemotherapy in metastatic melanoma management [45,47,48]. Other studies have shown that the combination of trametinib with dabrafenib is superior to chemotherapy in brain metastases (COMBI-D) or adjuvant therapy (COMBI-AD) [45,47,48].

##### Vemurafenib

Vemurafenib is indicated in monotherapy for the treatment of adult patients with BRAF V600 mutation-positive unresectable or metastatic melanoma [49]. The recommended dose is 960 mg (4 tablets of 240 mg) twice daily [49]. The BRIM3 study has reported that vemurafenib is superior to chemotherapy in patients that have been previously treated and in patients with metastatic melanoma that is BRAF V600E mutation positive [49].

##### Encorafenib

Encorafenib is indicated for use in combination with binimetinib for the treatment of adult patients with unresectable or metastatic melanoma with a BRAF V600 mutation [50]. It is also indicated for use in combination with cetuximab in metastatic colorectal cancer [50]. The recommended dose is 450 mg once daily, in combination with binimetinib [50].

##### Binimetinib

Binimetinib is indicated for use in combination with encorafenib for the treatment of adult patients with unresectable or metastatic melanoma with a BRAF V600 mutation [51]. The recommended dose is 45 mg (three 15 mg tablets) twice daily [51]. The CMEK16B2301 study has reported that the association of encorafenib with binimetinib is significantly superior regarding progression-free survival (PFS) compared with vemurafenib [51].

##### Cobimetinib

Cobimetinib is an MEK inhibitor licensed for the management of unresectable or metastatic melanoma with a BRAF V600 mutation in combination with vemurafenib, at the dosage of 60 mg/die [52]. A phase III study has shown that patients receiving cobimetinib plus vemurafenib had a statistically significant improvement of PFS as compared with patients receiving vemurafenib plus placebo [53].

#### 3.3.3. Oncolytic Virus Therapy

Talimogene laherparepvec (T-VEC) is an oncolytic immunotherapy derived from the herpes symplex virus type-1, indicated as being of use for the management of adults with an unresectable melanoma that is regionally or distantly metastatic (Stage IIIB, IIIC and IVM1a) with no bone, brain, lung or other visceral disease [54]. Its mechanism of action is based on its selective replication within tumors, causing an antitumor response thanks to the production of granulocyte macrophage colony-stimulating factor [55]. A phase II trial involving 50 patients with visceral metastases (*n* = 24), skin or lymph node metastases (*n* = 16) and unresectable regional disease (*n* = 10) receiving T-VEC Q3W reported an ORR of 26% [55,56]. Moreover, a phase III trial (NCT00769704) with 436 patients with unresectable melanoma randomized to receive intralesional T-VEC (*n* = 295) or granulocyte macrophage colony-stimulating factor (*n* = 141) showed a significant improvement in T-VEC cohort (26.4 vs. 5.7%, *p* < 0.0001) [57].

#### 3.3.4. Engineered Cytokines

Bempegaldesleukin is an engineered IL-2R agonist which acts by the reduction of IL-2 binding to CD25 over CD122/CD132, leading to an antitumor immune response [52]. A phase II trial (PIVOT-02), enrolling 38 patients with untreated metastatic melanoma, has reported an ORR of 52.6% after 29 months of therapy [58]. A phase III study (PIVOT IO 001) is ongoing [59].

Darleukin (L19IL2) is a fully human immunostimulatory product consisting of the fusion of the human L19 antibody and IL2 [60]. Its effectiveness has been investigated in a phase II study enrolling 69 subjects with metastatic melanoma treated with dacarbazine (*n* = 24) or dacarbazine plus darleukin at two dosages (*n* = 23 and 22, respectively) [54]. A significant ORR reduction was reported in patients receiving L19IL2 plus dacarbazine as compared with dacarbazine only [60]. A study (Neo-DREAM) evaluating the effectiveness of neoadjuvant intratumoral darleukin/fibromun (L19IL2 + L19TNF) in patients with stage IIIB/C melanoma is ongoing [60].

#### 3.3.5. Other Treatments

There are several treatments for CM management under investigation [58,60,61,62,63,64,65,66,67,68,69,70,71,72,73]. Among these, fighting the acquired resistance of immunotherapy and targeted therapy is one of the main aims. Among the proposed strategies, the sequential use or combination of targeted therapy and immunotherapy seems to be a valuable option, leading to the development of several studies which are ongoing. Other treatments have been proposed, such as drugs targeting inhibitory molecules (e.g., colony-stimulating factor 1 receptor inhibitors and indoleamine 2,3-dioxygenase 1 inhibitors), intravenous oncolytic virus (e.g., ICOVIR-5), toll-like receptor agonists, agonistic anti-OX40 antibodies, antivascular endothelial growth factor (e.g., bevacizumab), T-cell agonists, glucocorticoid-induced tumor necrosis factor receptor family-related protein and adoptive T-cell therapy. Moreover, new knowledge on melanoma pathogenesis has led to potential therapeutic strategies such as gene therapies or therapies against the tumor microenvironment. Similarly, nanotechnology may provide strategies for the targeted delivery of drugs, genes, and proteins to tumors. Finally, the use of radiotherapy cannot be undervalued. Indeed, radiation may increase tumor antigen visibility and promote the priming of T cells but can also exert immunosuppressive action on the tumor microenvironment. Thus, the combination of radiotherapy and immunotherapy may increase treatment outcomes.

During the COVID-19 pandemic [74,75,76], mRNA vaccines were rigorously studied, revealing the potential of this cutting-edge technology to innovate melanoma treatment. Experiments in animal models and clinical trials have shown promising results, creating a solid foundation for more systematic research in the years to come. In particular, the KEYNOTE-942 study, which combines an mRNA vaccine with an immune checkpoint inhibitor, aspires to achieve the next fundamental breakthrough in melanoma treatment.

Notably, recent knowledge on the role of melanin pigment in the defense against the damaging effects of ultraviolet radiation and other environmental stressors has led to the recognition of two different types of melanin: eumelanin, which is thought to offer radioprotection and photoprotection by functioning as an effective antioxidant and sunscreen, and pheomelanin which, being less photostable, can create a mutagenic environment after exposure to short-wavelength UV radiation [69,77,78,79]. In this context, melanogenesis, along with its highly reactive intermediates, can exhibit cytotoxic, genotoxic, and mutagenic activities [80,81,82]. This process can stimulate glycolysis and activate hypoxia-inducible factor 1-alpha (HIF-1α) [83]. Combined with their immunosuppressive effects, these factors can contribute to melanoma progression and resistance to immunotherapy [84,85,86]. Furthermore, the biophysical properties of melanin also render melanoma resistant to both chemotherapy and radiotherapy [87]. However, in vitro studies have shown that human melanoma cells containing melanin are less capable of spreading in nude mice compared with melanoma cells without the pigment, suggesting that the presence of melanin can inhibit the formation of melanoma metastases [88,89,90]. In summary, the Yin and Yang effects of melanogenesis should be considered in mechanism-oriented preclinical studies on melanomagenesis and melanoma progression, as well as in clinical efforts to manage this devastating disease for the benefit of patients, offering new therapeutic strategies.

Other emerging therapeutic options are related to recent knowledge on the cancer’s ability to disrupt local and systemic homeostasis [91]. Indeed, tumors are able to produce cytokines, immune mediators, neurotransmitters, hypothalamic and pituitary hormones, biogenic amines, melatonin, and glucocorticoids, which can influence key neuroendocrine centers such as the hypothalamus, pituitary, adrenals, and thyroid, thereby modulating body homeostasis via central regulatory axes [92,93,94]. Moreover, tumor-derived neurotransmitters may impact both body and brain functions [95,96]. As a consequence, cancers may have the ability to manipulate the central neuroendocrine and immune systems to reset body homeostasis in a manner that favors their own growth at the expense of the host. All of these pathways may be new therapeutic targets.

## 4. Expert Opinion

The recent knowledge on the pathogenesis of skin cancers has led to the development of effective and selective therapies [97,98,99,100,101,102,103,104].

Notably, the role of UV radiation has also been completely revised [105]. It is well-known that UV radiation is associated with negative effects, such as skin aging, cancer formation, and autoimmune conditions. However, the positive impact of UV radiation on homeostasis is often overlooked. Indeed, when skin is exposed to UV, it initiates local responses triggered by chemical, hormonal, immune, and neural signals, which are influenced by the types of chromophores present and the depth of UV radiation penetration into skin layers [106,107,108,109]. These responses are not random but are orchestrated by the cutaneous neuro-immuno-endocrine system, which counteracts external stressors and adapts local homeostasis to environmental changes [106,107,108,109,110]. Understanding the mechanisms of UVR penetration into the body and its impact on the brain and internal organs is crucial for providing insights for novel therapeutic approaches in conditions such as addiction, mood disorders, autoimmune diseases, neurodegenerative disorders, chronic pain syndromes, and pathologies involving the endocrine, cardiovascular, gastrointestinal, or reproductive systems.

Globally, the therapeutic landscape for advanced melanoma has completely changed over the past decade. Indeed, the advances in immunotherapy and targeted therapies have led to the development of selective and effective drugs. However, several challenges remain. Globally, the treatment of invasive melanomas is influenced by tumor thickness, lymph node involvement and the presence of metastasis. In this context, though in situ melanoma does not require further treatment except for a limited secondary excision, the biopsy of the sentinel lymph node is recommended for patients with a CM thicker than 1 mm or 0.8 mm with peculiar histological features. Patients with unresectable disease or lymph node involvement require a personalized approach.

In these cases, neoadjuvant treatments are under investigation. As regards adjuvant therapies, there are well-established guidelines for subjects with stage III/IV melanoma. However, a subgroup of patients with stage II melanoma may also benefit from these treatments, in order to reduce the risk of tumor recurrence. Emerging therapies and technologies, as well as new prognostic data on CM, have raised several controversies surrounding the existing staging system. Among these, the debate around the importance of SNLB should be underlined. Indeed, recent studies have evaluated the use of tumor histopathologic characteristics, specific patient populations, and gene expression profiling to predict SLN positivity, shedding light on the possibility of SLN biopsy omission.

Several clinical trials have reported the effectiveness and safety of these treatments. Currently, drugs such as nivolumab, pembrolizumab (which block the PD-1/PD-L1 pathway), and ipilimumab (targeting CTLA-4) form the cornerstone of immunotherapy. Meanwhile, targeting the BRAF V600 mutation with drugs like dabrafenib, vemurafenib, and encorafenib, as well as MEK1 and MEK2 with trametinib, constitutes the primary approach in targeted therapy.

Moreover, the association of immune checkpoint inhibitors and targeted therapies in the management of metastatic melanoma may represent a breakthrough in the treatment scenario of advanced melanoma, increasing the therapeutic effectiveness. Finally, one should underline the elevated level of safety of these drugs, which have completely replaced classical chemotherapy. However, there are still patients that do not receive clinical advantages from these therapies, leading to the need for new strategies. Indeed, CM has the capacity to develop several mechanisms to gain resistance to targeted therapy (e.g., re-activation of the MAPK pathway, activation of substitutive pathways, changes in tumor microenvironment, miRNA-mediated resistance mechanisms, etc.) and to ICI.

In this context, several new drugs with different mechanisms of action such as intravenous oncolytic virus (e.g., ICOVIR-5), antivascular endothelial growth factor (e.g., bevacizumab), drugs targeting inhibitory molecules (e.g., colony-stimulating factor 1 receptor inhibitors and indoleamine 2,3-dioxygenase 1 inhibitors), T-cell agonists, toll-like receptor agonists, agonistic anti-OX40 antibodies, glucocorticoid-induced tumor necrosis factor receptor family-related protein and adoptive T-cell therapy, are under investigation. Furthermore, radiotherapy also seems to have a possible role in melanoma management. In our opinion, the introduction of these therapies may open new scenarios for those patients who do not respond to immune checkpoint inhibitors and targeted therapies. Moreover, newer technologies, such as nanotechnology, may provide strategies for the targeted delivery of drugs, genes, and proteins to CM.

Medicine is moving towards an ever more personalized approach, and more knowledge of melanoma pathogenesis and on its mechanisms of immune escape will allow the development of selective and effective drugs, specifically targeting the tumor environment and sparing healthy cells. In the future, the management of AM will change, and effective and safe drugs will be available. Indeed, biomarkers and tumor microenvironment parameters will have increasing importance in clinical decision making and offer a more precise and personalized approach.

However, in our opinion, the challenge for the treatment of melanoma is still to be resolved. Nevertheless, we are moving towards increasingly specific and detailed therapies tailored for each patient depending on their anamnesis, a tailored-tail approach will increase survival in AM management.

## 5. Conclusions

The therapeutic scenario of melanoma management is changing. Despite the various treatments that are available, there is still the need for new treatments for certain stages of melanoma or for melanoma that is unresponsive to currently available therapeutic options. Therefore, new studies are necessary to identify new treatment options and to better use currently available drugs.

## Figures and Tables

**Table 1:** Currently approved drugs for advanced melanoma.

Drug	Type of Drug	Mechanism of Action
Nivolumab	Immune checkpoint inhibitors	PD-1 antagonist
Pembrolizumab	Immune checkpoint inhibitors	PD-1 antagonist
Ipilimumab	Immune checkpoint inhibitors	CTLA-4 antagonist
Nivolumab + Ipilimumab	Immune checkpoint inhibitors	PD-1 + CTLA-4 antagonist
Dabrafenib	Kinase inhibitor	Mutant BRAF
Trametinib	Kinase inhibitor	MEK
Vemurafenib	Kinase inhibitor	Mutant BRAF
Encorafenib + Binimetinib	Kinase inhibitor	Mutant BRAF and MEK
Trametinib + Dabrafenib	Kinase inhibitor	MEK and mutant BRAF
Cobimetinib + Vemurafenib	Kinase inhibitor	MEK and mutant BRAF
Talimogene laherparepvec	Oncologic virus	Virus-mediated GM-CSF production
Dacarbazine	Antineoplastic	Anti-mitotic and anti-metastatic
Aldesleukin	Cytokine	IL-2/IL-2R pathway
Peginterferon Alfa-2b	Cytokine	IFNAR1/2 pathway
High-dose Interferon Alfa-2b	Cytokine	IFNAR1/2 pathway

## Data Availability

The authors confirm that the data supporting the findings of this study are available within the article.
